# Health Literacy and Plan Choice: Implications for Medicare Managed Care

**DOI:** 10.3928/24748307-20180201-01

**Published:** 2018-03-09

**Authors:** Robert T. Braun, Andrew J. Barnes, Yaniv Hanoch, Alex D. Federman

## Abstract

**Background:**

Older adults with inadequate health literacy may have difficulty selecting optimal coverage when faced with multiple health insurance plans to choose from.

**Objective:**

This study sought to examine how health literacy affects which Medicare Advantage plans seniors select.

**Methods:**

We surveyed 311 Medicare beneficiaries who did not have concurrent Medicaid coverage. Participants chose from three Medicare Advantage plans: (1) lower-premium, less coverage; (2) higher-premium, more coverage; and (3) an intermediate option. Adjusted associations between health literacy, plan choice, and the importance of plan attributes in decision-making were tested using ordered and multinomial logistic regressions.

**Key Results:**

Beneficiaries with inadequate health literacy chose the lower-premium, less coverage plan over the higher-premium, more coverage option compared to beneficiaries with adequate health literacy (*p* < .05) perhaps because participants with inadequate health literacy tended to rank the importance of plan attributes differently than those with adequate health literacy (*p* < .05).

**Conclusions:**

This evidence suggests there may be a disconnect among those with inadequate health literacy between attributes that were ranked as important and the plans they chose, resulting in choices that are not consistent with their preferences. Beneficiaries with inadequate health literacy may be at increased risk of selecting plans that do not meet their health needs, resulting in reduced access and higher costs. Medicare and consumer groups should support interventions to raise literacy levels and those that reduce the reliance on literacy when plan shopping. **[*HLRP: Health Literacy Research and Practice*. 2018;2(1):e40–e54.]**

**Plain Language Summary:**

We examined how health literacy affects seniors' choice of Medicare Advantage plans. Results indicate that beneficiaries with inadequate health literacy chose the lower-premium, less coverage plan over the higher-premium, more coverage option compared to beneficiaries with adequate health literacy. Beneficiaries with inadequate health literacy tended to rank the importance of plan attributes differently than those with adequate health literacy.

Medicare beneficiaries now face an unprecedented array of privately managed health insurance options. These include stand-alone Medicare Part D prescription plans, with dozens of options available in most markets, and Medicare Advantage plans, of which 1 to 6 options may be available (Gold, 2009; Kaiser Family Foundation, 2013). Adding to this complexity, the Medicare Advantage plans include traditional managed-care plans, private fee-for-service plans, and preferred provider organization plans. Medicare beneficiaries can also supplement traditional fee-for-service Medicare with privately purchased Medigap coverage, and they may qualify for a variety of low-income assistance programs. Although the health care system is currently in a state of flux, it is doubtful that Medicare coverage-related decisions will become simpler or easier to make.

Choosing from so many options can be challenging for any adult, but older adults in general have a greater disadvantage because of their complicated financial and health circumstances (e.g., greater risk of poor health outcomes, limited life span, and fixed incomes), problems accessing information, and cognitive issues, such as reduced processing speed and working memory (Hibbard, Jewett, Engelmann, & Tusler, 1998; Kaiser Family Foundation, 2012; Salthouse, 1996). Moreover, prior research has suggested that difficulty comprehending health insurance or low health insurance literacy—as a subcomponent of health literacy in general—may contribute to a disconnect between what people say they want from an insurance product and what they end up choosing (Barnes, Hanoch, & Rice, 2015; Federman, Safran, Keyhani, Siu, & Halm, 2008; Paez et al., 2014). This is consistent with additional research that suggests that older adults seek less information to help with decision-making (Finucane et al., 2002; Peters, Diefenbach, Hess, & Västfjäll, 2008), take less time deciding, make less complex comparisons (Kim, Goldstein, Hasher, & Zacks, 2005), and have a greater reliance on shortcuts and heuristics (Federman et al., 2008; Finucane et al., 2002). Furthermore, cognitive ability closely relates to health literacy, as health literacy draws on working memory and executive function (Federman et al., 2008; Wolf et al., 2012).

Health literacy is defined as the ability to comprehend basic health information and to make suitable health-related decisions (Barnes, Hanoch, & Rice, 2015; Koh & Rudd, 2015; Paez et al., 2014; Wizemann, 2011), but how health literacy relates to insurance choices, particularly among seniors, is poorly understood. Because inadequate health literacy and insurance decision-making complexity are particular concerns for Medicare beneficiaries, a better understanding of how the two relate is essential to improve the presentation of coverage choices to older adults (i.e., choice architecture) and to educate older adults about choosing and using Medicare coverage more effectively. In this study, we examined the associations of health literacy with hypothetical Medicare Advantage plan choices and the importance of various plan attributes for plan choices among community-residing, lower income, older adults in New York City. We hypothesized that beneficiaries with inadequate health literacy would more likely choose plans offering them less protection, in part because they would place less importance on the depth of coverage for a given plan (e.g., out-of-pocket maximum, out-of-network access) and more importance on “prices” (e.g., premiums, copays) than those with adequate health literacy.

## Methods

### Subjects and Settings

We recruited independently living, low-income adults, age 60 years and older, from 30 community-based settings in Manhattan, including senior centers and residential complexes. We identified senior centers, either free-standing or connected with naturally occurring retirement communities, through the listings of the New York City Department for the Aging. We identified low-income housing facilities from a listing of federal Housing and Urban Development supported low-income housing facilities. We selected sites in zip code areas with median household incomes below $50,000. We oversampled men because women outnumber them in these communities. In total, 456 participants responded to the survey. Of these, we excluded 145 from our analysis because they reported having both Medicaid and Medicare coverage. This analysis focused on the subgroup of Medicare beneficiaries who did not have concurrent Medicaid coverage (*N* = 311). We excluded these beneficiaries because we wanted to examine people who had only Medicare coverage. Furthermore, people who enroll in Medicare and Medicaid tend to have different health needs than beneficiaries with just Medicare.

Recruitment at each site followed rules set forth by the site's management staff but occurred mostly during site-provided meals and site-sponsored special events. We recruited people for a longitudinal study about health, health care use, and health insurance that provided $20 for the baseline interview and $10 for a follow-up interview (scheduled to occur 6–9 months later). We conducted interviews with only a single member of a household, on site, in English or Spanish, and by trained bilingual interviewers. The Icahn School of Medicine at Mount Sinai Institutional Review Board approved this study.

### Plan Choice and Importance of Plan Attributes in Decision-Making

**Figure 1.**
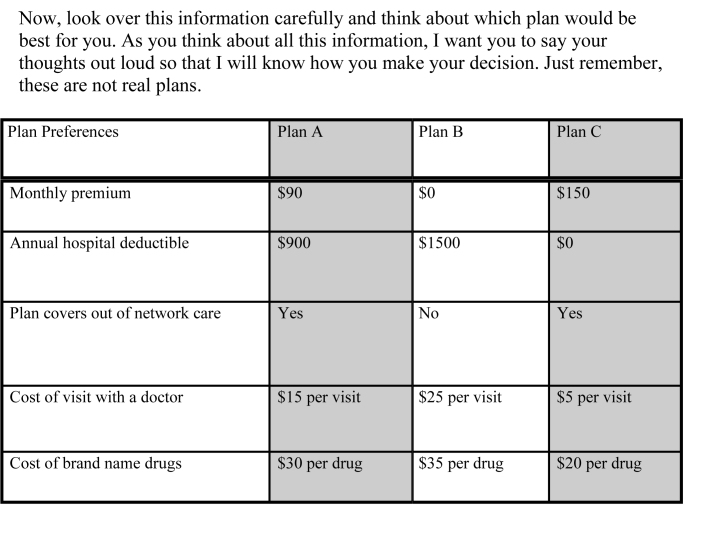
Hypothetical Medicare Advantage Plan choice task.

The first outcome of interest was beneficiaries' choice from among three hypothetical plans similar to options in the Medicare Advantage market (**Figure 1**). Each plan consisted of five attributes: monthly premium, hospital deductible, outpatient visit copayments, prescription drug copayments, and coverage for out-of-network care. The three plan choices offered a range of coverage options: lower-premium, less coverage option (labeled as Plan B in **Figure 1**); a higher-premium, more coverage option (Plan C); and an intermediate option (Plan A). Coverage for outof-network care, visits, and brand-name drug copayment options also varied.

The second outcome of interest was participant rankings of the relative importance of the five plan attributes: low copayments for outpatient visits, low copayments for brand-name drugs, low monthly premiums, no deductible for hospital stays, and coverage for out-of-network physicians (**Table A**). To avoid biasing the participants' choices during the attribute importance ranking exercise, we printed the five plan attributes on individual strips of paper and asked study participants to rank them by positioning the strips in order of importance.

### Health Literacy

We measured health literacy using the Short Test of Functional Health Literacy in Adults (S-TOFHLA). Scores on the S-TOFHLA indicate three levels of health literacy: adequate, marginal, and inadequate (Gazmararian et al., 1999). People with inadequate health literacy struggle with basic medical information, such as reading prescription bottles (Gazmararian et al., 1999). S-TOFHLA is a shortened version of the Test of Functional Health Literacy in Adults, which takes an estimated 12 minutes to administer and is a valid and reliable instrument to identify people with deficits in understanding health information (Gazmararian et al., 1999).

### Demographics and Health Status

We also collected several control variables and included them in the analysis. These included demographic characteristics: age (60–69, 70–79, 80+ years), gender, race (White, Black, other), education (less than high school, high school, some college, or more), monthly income (≤$750, $751–$2,000, >$2,000), and current insurance type (Medicare fee-for-service, Medicare Advantage, other, supplemental Medicare Part D prescription drug coverage, Medigap). We measured general health status by patients self-reporting how they rate their general health. Patients answered poor, fair, good, very good, or excellent. We then collapsed general health status into three categories (excellent/very good, good, fair/poor). We measured chronic disease by summing up whether a patient reported asthma, diabetes, depression, anxiety, osteoporosis, congestive heart failure, cancer, hyper-tension, gastroesophageal reflux disease, emphysema, and/or arthritis. We then categorized the summed value (0–1 conditions, 2–3 conditions, and >4 conditions). We assessed functional status for six activities of daily living (ADL), including getting into or out of bed or chairs, walking, eating, dressing, bathing, and toileting. We used instrumental activities of daily living (IADL) to measure functional status by assessing whether patients could do light or heavy housework, prepare their own meals, shop for personal items, and pay their bills and track expenses. Respondents reported whether they had no, a little, some, or a lot of ability to do each of the ADLs or IADLs we assessed (Linn & Linn, 1982). We considered study participants impaired for an ADL and/or IADL if they had difficulty with one or more tasks “a lot” of the time or could not perform them (Linn & Linn, 1982). To measure utilization, we included the number of medications, visits to the emergency department (ED), and admissions in the models. Patients self-reported visits to the ED (0–1, 2–5, 6+) and admissions (0–1, 2–5, 6+) to the interviewer. Patients brought their medications or medication lists to the interviews. We then summed the number of medications and categorized it into three categories (0–2, 3–6, 7+). We also assessed health care use in the past year including patients' self-reported number of primary care visits (0–1, 2–3, 4–5, 6+) and specialty care visits (0–3, 4–8, 9+). To measure beneficiary medical costs, we introduced self-reported monthly medication costs ($0–9, $10–49, $50+), and whether the beneficiary reported having trouble paying medical costs (*not at all*, *somewhat*, *difficult*, *very difficult*) into the models.

### Statistical Analysis

Item nonresponse rates for the 311 Medicare beneficiaries who did not have concurrent Medicaid coverage ranged from 1% to 14% (**Table B**). Of the 311 survey respondents, 219 (70%) completed the survey questions in full, whereas 14% were not able to complete the plan choice exercise and 6% were not able to complete the S-TOFHLA. Of those with missing plan choice information, those with poor health literacy had the highest in percent as a group (20%). Adequate and marginal literacy respondents had roughly the same percent (7%) missing information. We assumed that data were missing completely at random, and we used a multiple imputation by chained equation approach to impute 20 complete data sets that we combined and used in all analyses (Allison, 2001; Royston, 2009). We used chi-square tests (χ^2^) to test for unadjusted associations between health literacy levels, plan choice, and plan attribute importance rankings. We ran a multinomial logistic regression model to examine whether plan choices varied across health literacy levels after controlling for patient characteristics. Postestimation, we also converted associations between health literacy and plan choices to probabilities for ease of interpretation. To estimate the adjusted association between plan importance rankings and health literacy, we fitted an ordered logistic regression to the data after confirming the assumption of proportional odds. In both regression models, we used robust standard errors. We reported relative risks (RR) for multinomial logistic regression models, and we reported odds ratios for ordered logistic regression models. We set the probability of making a type II error to 5% for all significance tests. We conducted all analyses using Stata Statistical Software 14.

## Results

### Descriptive Statistics

Most Medicare beneficiaries participating in the study (63.4%) selected the higher-premium, more coverage plan choice (Plan C), followed by the intermediate option (Plan A; 27%), and the lower-premium, less coverage plan choice (Plan B; 9.6%; **Table 1**). Most (72.8%) Medicare beneficiaries reported adequate health literacy. Participants tended to be age 70 to 79 years (37.7%), female (76.2%), and White (38.5%); they had at least some college education (57.9%) and had monthly incomes of $751–$2,000 (52.3%). Many participants reported fair or poor health (33.2%) and had deficiencies in IADLs (66.2%) or ADLs (43.2%). A range of beneficiaries reported having 2 to 3 co-occurring chronic conditions (46.2%), taking 3 to 6 prescriptions daily (45.1%), and spending between $10 and $49 monthly on medications (36.5%). Most beneficiaries had one or no ED visits (62.5%) and admissions (77.5%) to the hospital in the past year, whereas 18.2% of beneficiaries reported having a difficult or very difficult time paying their health care costs.

### Health Literacy and Medicare Advantage Plan Choice

Prior to adjustment for participant characteristics, there were no significant bivariate associations between health literacy and plan choice. However, after adjustment for participant demographics (age, gender, race, education, monthly income, insurance type), health status (general health status, number of chronic diseases, ADLs, IADLs), and past year health services use (number of medications, visits to the ED, number of admissions to hospital, primary care visits, specialty care visits) and medical costs (monthly medication costs, having trouble paying medical bills), Medicare beneficiaries with inadequate health literacy were more likely to choose the lower-premium, less coverage option (Plan B) over the higher-premium, more coverage option (Plan C) than beneficiaries with adequate literacy (RR = 5.58; 95% confidence interval [CI] 1.12–27.90; **Table 2**). There was no difference in plan choice between beneficiaries who had adequate or marginal literacy. When we converted to predicted probabilities, beneficiaries with adequate health literacy had a much higher probability of selecting Plan C, the higher-premium, more coverage option, (68%, *p* < .05) than people with inadequate health literacy (52%, *p* < .05; **Table C**). Moreover, beneficiaries with inadequate health literacy had a higher probability of selecting Plan B, the lower-premium, less coverage option, (24%, *p* < .05) than beneficiaries with adequate health literacy (8%, *p* < .05). Beneficiaries with marginal literacy had a similar trend to those with adequate literacy, unlike those with inadequate literacy. Beneficiaries with marginal literacy had a much higher probability of selecting Plan C, the higher-premium, more coverage option (67%, *p* < .05) and did not differ significantly in choosing Plan B, the lower-premium, less coverage option (5%, *p* > .05).

### Beneficiaries' Rankings of the Importance of Plan Attributes in Decision-Making

All participants ranked plan attributes and placed them in ascending order. Access to out-of-network providers (49%) and having no hospital deductible (36%) were more important than low office visit copayments (5%), brand-name drug fees (5%), and monthly premiums (6%). Before adjustment for participant characteristics, participants' rankings of the importance of plan attributes varied significantly across their level of health literacy (*p* < .01). Specifically, more participants with adequate health literacy ranked out-of-network provider access as the most important factor in choosing a health plan (55%) than participants with marginal (35%) or inadequate (27%) health literacy. Conversely, participants with adequate health literacy were less likely to rank low office visit copays as the most important factor (2%) than participants with marginal (4%) or inadequate (15%) health literacy.

After adjusting for participant characteristics, health literacy had a significant association with the importance ranking of plan attributes (**Table 3**). In particular, participants with inadequate health literacy reported lower odds of ranking no hospital deductible and access to out-of-network providers as more important than having lower premiums and lower co-pays for branded drug and office visits than people with adequate health literacy (odds ratio = 0.32, 95% CI 0.13–0.82). A similar pattern emerged for participants in the middle-income range, unlike those in the highest income range and male participants *(p* < .05). Conversely, beneficiaries with current supplemental drug or gap coverage and those with any IADL impairment had higher odds of ranking no hospital deductible and access to out-of-network providers as more important (*p* < .05 each).

### Sensitivity Analyses

We investigated the robustness of our health literacy estimates by collapsing the marginal and inadequate levels and re-estimating our models. Overall, the results (**Table D**) across all models indicate a similar pattern of adjusted associations between the collapsed health literacy measure, plan importance rankings, and plan choice, although the magnitudes of the estimates were smaller and the confidence intervals were less compact. We also tested the sensitivity of our imputation approach to complete case analysis and found the health literacy estimates using imputation were more conservative and had similar precision to fitting models to data from only the complete cases.

## Discussion

As the growth in Medicare Advantage plans continues, beneficiaries will increasingly face a range of privately managed insurance options, so they will need to understand and evaluate plan differences, and to choose plans that best suit their needs. To our knowledge, this is the first study examining the extent to which Medicare beneficiaries trade off premiums for coverage, and the insurance plan attributes Medicare beneficiaries consider most important in their plan choices, which vary across beneficiaries' level of health literacy.

Evidence from our study suggests that beneficiaries with inadequate health literacy were much more likely to choose lower cost, lower coverage plans over plans offering more risk protection for a higher premium than beneficiaries with adequate literacy. Moreover, beneficiaries with inadequate health literacy appear different from those with adequate and marginal health literacy in the plan attributes they give the most weight. Yet, participants with inadequate health literacy were less likely to choose plans that aligned with their own stated importance rankings. For example, those with inadequate health literacy place more importance on low copays for office visits yet were more likely to choose the plan with the highest copays. Taken together, our results suggest that Medicare beneficiaries with inadequate health literacy may be less likely to understand plan features relating to the depth of coverage and thus may be particularly disadvantaged when shopping in Medicare Advantage and stand-alone Part D markets. Moreover, beneficiaries with poor health literacy were more likely to be missing plan choice information compared to those with adequate health literacy. Thus, the estimates presented in **Table 2** likely understate the relationship between beneficiaries having poor health literacy and selecting the lower cost, lower coverage plan.

### Study Limitations

This study has limitations. First, the sample was one of convenience and our sample of elderly, New York community-dwelling residents may not generalize to all Medicare beneficiaries. However, the distribution of health literacy levels of the participants is similar to national estimates from the 2003 National Assessment of Adult Literacy report (Kutner, Greenburg, Jin, & Paulsen, 2006). Additionally, the prevalence of inadequate health literacy in our sample is consistent with earlier work (Gazmararian et al., 1999). Second, the plan choice task was hypothetical, with no financial or health impact on the participants. However, evidence from other experiments in which people received incentives to choose the plan with the lowest total costs indicates that decision errors are common, even when money is at stake (Barnes, Hanoch, & Rice, 2016), a finding echoed by research from the employer-sponsored market, where many employees choose plans that are inferior to other choices available to them (Bhargava, Loewenstein, & Sydnor, 2015). Further, given that beneficiaries shopping for plans in Medicare Advantage markets face, on average, 1 to 6 plans to pick from, and dozens of stand-alone Medicare prescription drug plans, our study's finding that those with inadequate health literacy experience difficulty choosing from three simplified plan options is especially concerning, and probably conservative by nature (Gold, 2009; Kaiser Family Foundation, 2013).

The insurance decision task asked participants to make choices without decision support and did not examine whether the decision-making strategies and the quality of decisions among beneficiaries with inadequate health literacy would improve had they received the assistance that many organizations offer seniors. It is also unclear whether beneficiaries with inadequate health literacy use such decision-support opportunities (Goldstein, Teichman, Crawley, & Gaumer, 2001). Nonetheless, with the increasing role of Medicare Advantage plans in the Medicare market, Centers for Medicare and Medicaid Services (CMS) has made significant efforts to inform beneficiaries better of their plan benefits and choices, and it uses a 5-star rating system, which provides quality ratings of Medicare Advantage plans (Darden & McCarthy, 2015). However, there is limited evidence that the CMS Medicare Advantage plan rating system has succeeded in steering beneficiaries toward more highly rated plans (Darden & McCarthy, 2015). Finally, our analyses did not include measures of cognitive performance, which may have additionally affected how people prioritized health plan choices.

## Conclusions

These results add to the growing literature on the importance of inadequate health literacy as a contributor to adverse health outcomes and excessive medical costs, and as a main driver of health disparities (Peters, Hibbard, Slovic, & Dieckmann, 2007). Furthermore, the reluctance of Medicare Advantage and Medicare prescription drug plan beneficiaries to switch health plans compounds inadequate health literacy and poor plan selections. From 2013 to 2014, 78% of Medicare Advantage enrollees stayed in their plans, and only 1 in 10 enrollees switched plans (Jacobson, Neuman, & Damico, 2016). Most beneficiaries who enrolled in a lower quality-rated Medicare Advantage plan remained in that plan, whereas 14% voluntarily switched to a plan with only modestly higher quality ratings (Jacobson et al., 2016). Furthermore, the stationary behavior of Medicare Advantage beneficiaries increased their medical cost and plan costs. On average, a Medicare Advantage beneficiary who switched plans may have saved $17.51 a month in premiums and lowered their out-of-pocket maximums by $401 (Jacobson et al., 2016).

The present study demonstrates that Medicare beneficiaries with inadequate health literacy, on average, are not selecting plans based on their health preferences, and are less likely to discern fundamental cost-sharing features of their plan. These health insurance decision errors arise from such Medicare beneficiaries having difficulty obtaining, understanding, and using information about how plan choices differ. These frictions can result in inconsistent plan choices where stated preferences (what consumers say they want) and revealed preferences (what they actually choose) do not align (Barnes, Hanoch, & Rice, 2015). This difficulty understanding plan information hampers plan choices that affect the individual and are problematic in a market environment that presupposes that consumers shop by making well-informed comparisons of costs and quality of health plans (Gruber, 2009; Jewett & Hibbard, 1996; Kutner et al., 2006). Addressing health insurance literacy deficits is an important aspect of general health literacy. Educating seniors about insurance plan features provides one avenue to improve the quality of their coverage choices. Moreover, policies to improve health insurance literacy should also guide particularly vulnerable beneficiaries, such as the elderly, to make better plan choices by requiring simple and yet more thoughtful presentation of information for them to compare Medicare Advantage plans more easily.

It should also be noted that our findings should not be considered as characterizing older beneficiaries alone, as a growing body of work (Loewenstein et al., 2013) demonstrate that individuals of all ages lack knowledge about health insurance and are often making less than ideal choices. For example, work by Barnes, Hanoch, Rice, and Long (2017) reported that individuals often choose their insurance based on their monthly premium rather than total estimate cost. They also found that making simple alterations and providing additional information about health insurance terminology did improve participants' decisions.

## Practice Implications

Traditional methods of presenting insurance plan options are often not tailored for senior (or other) citizens; in particular, although the National Assessment of Adult Literacy has shown repeatedly that the majority of Americans older than age 65 years have “basic” or “below-basic” document literacy (the ability to interpret discontinuous text, such as graphs and charts), insurance plan option presentations are most often in chart-form (Darden & McCarthy, 2015). In line with prior work, our study further highlights the need to address health literacy as a national problem, with serious financial and health ramifications for millions of people. Our investigation also used a table (albeit a simpler one than those on the Medicare website) to compare health insurance options. Whether seniors would be able to navigate the same information better if providers presented it in alternative forms, and whether this might in fact alter their decision-making process, requires further study. Alternative forms may include guided-decision models (Hibbard, Slovic, & Jewett, 1997; Leonard, 2008; Wood et al., 2011) that highlight similarities and differences among plans, so that consumers can identify a few priorities and then select a plan that emphasizes those elements. Some people may also face disadvantages in their understanding of health plan information due to lack of experience of purchasing a plan or accessing care (Jewett & Hibbard, 1996).

Another potential component for guided-decision models might be prompts that elicit patient experiences and preferences explicitly and then match those elements to plans that theoretically fit their needs. Another possibility is to educate consumers about health insurance, providing them with better insights about the possible tradeoffs between premium and deductible. Such an approach has already received some supporting evidence, albeit with nonelderly populations (Barnes et al., 2017). Moreover, policymakers might consider standardizing the presentation of Medicare Advantage plans by providing guidance on how to present plan attributes and options. In summary, our results suggest that policies aimed at changing the choice environment—via reduction of number of choices or tailoring the information to suit seniors better—could have financial and health benefits for consumers, particularly those with lower levels of health literacy, and public payers in the Medicare Advantage and stand-alone Part D markets.

## Figures and Tables

**Table 1:** Participant Characteristics (*N* = 100)

**Variable**	**Percentage**

Plan choice	
Plan A	27
Plan B	9.6
Plan C	63.4
Most important plan attribute	
Network provider access	48.8
No hospital costs	36.3
Low monthly cost	5.5
Low fees for brand name prescription	5.2
Low office visit fees	4.2
Health literacy	
Adequate	72.8
Marginal	9.3
Inadequate	17.9
Age	
60–69 years	31.6
70–79 years	37.7
80+ years	30.7
Sex	
Male	33.8
Race	
White	38.5
Black	32.9
Other	28.6
Education	
Less than high school	20.2
High school	21.9
>High school	57.9
Monthly income	
Low income (≤$750)	9.5
Middle income ($751–$2,000)	52.3
High income (>$2,000)	38.2
Insurance	
Medicare Advantage	33.6
Medicare FFS	11.2
Supplemental (MediGap)	31.7
Supplemental prescription	17.3
Other	6.2
Health status	
Excellent/very good	29.8
Good	37
Fair/poor	33.2
Chronic conditions	
0–1	26.1
2–3	46.2
4+	27.6
Functional health status	
Any IADL impairment	66.2
Any ADL impairment	43.2
Annual primary care visits	
0–1	15.1
2–3	34.4
3–5	30.2
6+	20.3
Annual specialty care visits	
0–3	37.2
4–8	38.1
9+	24.7
Number of medications taken daily	
0–2	34.1
3–6	45.1
+7	20.8
Monthly medication costs	
$0–$9	28.6
$10–$49	36.5
$50+	34.9
Annual emergency department visits	
0–1	62.5
2–5	34.3
6+	3.3
Annual admissions to hospital	
0–1	77.5
2–5	20.9
6+	1.6
Difficulty paying health care costs	
Not at all	63.2
Somewhat	18.5
Difficult	6.1
Very difficult	12.2

Note. ADL = activities of daily living; FFS = fee for service; IADL = instrumental activities of daily living.

**Table 2:** Adjusted Associations Between Medicare Advantage Plan Choice and Health Literacy (*N* = 311)

	**Medium Cost, Medium Coverage vs. Higher Premium, More Coverage (Plan A vs. Plan C)**	**Lower Premium, Less Coverage vs. Higher Premium, More Coverage (Plan B vs. Plan C)**

**Variable**	**Relative Risk [95% CI]**	

Education		
Less than high school	1.44 [0.65, 3.21]	1.19 [0.31, 4.54]
High school	1.58 [0.48, 5.24]	0.17^*^ [0.02, 1.24]
>High school	—	—

Monthly income		
Low income (≤$2,000)	1.06 [0.49, 2.29]	1.26 [0.37, 4.34]
High income (>$2,000)	—	—

Insurance		
Medicare FFS	0.82 [0.31, 2.18]	0.33 [0.06, 1.73]
Medicare Advantage	—	—
Supplemental prescription	0.78 [0.36, 1.71]	0.16^**^ [0.03, 0.93]
Supplement (MediGap)	1.36 [0.35, 5.33]	0.24 [0.01, 4.35]
Other	0.53 [0.15, 1.90]	0.63 [0.11, 3.74]

Health status		
Excellent/very good	—	—
Good	1.12 [0.48, 2.60]	0.34 [0.09, 1.34]
Fair/poor	1.2 [0.42, 3.44]	0.98 [0.20, 4.76]

Chronic conditions		
0–1	—	—
2–3	0.85 [0.37, 1.95]	1.36 [0.39, 4.74]
4+	0.97 [0.34, 2.80]	0.85 [0.14, 5.22]

Functional health status		
Any IADL impairment	1.93 [0.83, 4.48]	1.7 [0.42, 6.88]
Any ADL impairment	1.39 [0.63, 3.08]	2.89 [0.77, 10.86]

Annual primary care visits		
0–1	—	—
2–3	2.17 [0.75, 6.30]	0.67 [0.13, 3.42]
4–5	1.05 [0.33, 3.28]	0.76 [0.14, 4.08]
6+	1.18 [0.32, 4.29]	0.51 [0.06–4.54]

Annual specialty care visits		
0–3	1.57 [0.63, 3.95]	2 [0.48, 8.36]
4–8	—	—
9+	1.19 [0.57, 2.46]	2.01 [0.52, 7.87]

Number of medications taken daily		
0–2	—	
3–6	1.1 [0.50, 2.43]	1.63 [0.40, 6.58]
7+	0.88 [0.31, 2.49]	1.13 [0.16, 8.08]

Monthly medication costs		
$0–$9	—	—
$10–$49	0.84 [0.34, 2.05]	0.54 [0.13, 2.21]
$50+	0.72 [0.26, 1.97]	1.03 [0.22, 4.92]

Annual emergency department visits		
0–1	—	—
2–5	0.61 [0.30, 1.23]	0.74 [0.22, 2.54]
6+	0.57 [0.08, 4.00]	7.25^*^ [0.71, 73.62]

Annual admissions to hospital		
0–1	—	—
2–5	1.08 [0.46, 2.54]	2.88^*^ [0.88, 9.41]
6+	5.18 [0.43, 61.83]	0.01^**^ [0.00, 0.01]

Difficulty paying health care costs		
Not at all	—	—
Somewhat	0.72 [0.31, 1.68]	1.4 [0.37, 5.32]
Difficult	2.51 [0.78, 8.03]	2.3 [0.19, 27.20]
Very difficult	1.44 [0.43, 4.86]	3.5 [0.76,16.13]

Constant	0.08^***^ [0.01, 0.60]	0.02^***^ [0.00, 0.60]

Note. Adjusted associations between health literacy and Medicare Advantage plan choice were estimated using multinomial logistic regression. Plan C is the comparison choice. ADL = activities of daily living; CI = confidence interval; FFS = fee for service; IADL = instrumental activities of daily living.

* *p* < 0.1.

** *p* < .05.

*** *p* < .01.

**Table 3:** Adjusted Associations of the Importance of Plan Attributes in Decision-Making (*N* = 311)

**Variable**	**Odds Ratio [95% CI]**

Health literacy	
Adequate	—
Marginal	0.63 [0.25,1.56]
Inadequate	0.34^**^ [0.14, 0.81]
Age	
60–69 years	1.39 [0.72, 2.68]
70–79-years	0.83 [0.41, 1.67]
80+ years	—
Sex	
Male	0.48^**^ [0.26, 0.86]
Race	
White	—
Black	0.53^*^ [0.27, 1.06]
Other	0.59 [0.30, 1.15]
Education	
Less than high school	0.74 [0.36, 1.52]
High school	1.52 [0.59, 3.91]
>High school	—
Monthly income	
Low income (≤$2,000)	0.46^**^ [0.24, 0.87]
High Income (>$2,000)	—
Insurance	
Medicare FFS	3.82^***^ [1.62, 9.00]
Medicare Advantage	—
Supplemental prescription	3.35^***^ [1.70–6.60]
Supplemental (MediGap)	4.51^***^ [1.73,11.74]
Other	2.87^**^ [1.15, 7.17]
Health status	
Excellent/very good	—
Good	0.72 [0.37, 1.39]
Fair/poor	0.67 [0.31, 1.48]
Chronic conditions	
0–1	—
2–3	0.6 [0.31,1.19]
4+	0.89 [0.38, 2.10]
Functional health status	
Any IADL impairment	0.55^*^ [0.28, 1.11]
Any ADL impairment	0.93 [0.48,1.78]
Annual primary care visits	
0–1	—
2–3	1.24 [0.51, 3.00]
4–5	1.49 [0.58, 3.81]
6+	1.65 [0.59, 4.62]
Annual specialty care visits	
0–3	0.89 [0.45,1.79]
4–8	—
9+	1.12 [0.60, 2.11]
Number of medications taken daily	
0–2	—
3–6	0.88 [0.44, 1.72]
7+	1.31 [0.52, 3.28]
Monthly medication costs	
$0–$9	—
$10–$49	1.21 [0.61, 2.38]
$50+	0.95 [0.43, 2.12]
Annual emergency department visits	
0–1	—
2–5	0.91 [0.49, 1.67]
+6	0.54 [0.13, 2.32]
Annual admissions to hospital	
0–1	—
2–5	0.93 [0.49, 1.76]
6+	0.12^*^ [0.01, 1.07]
Difficulty paying health care costs	
Not at all	—
Somewhat	0.62 [0.34, 1.14]
Difficult	0.85 [0.23, 3.15]
Very difficult	0.48 [0.20,1.17]
Constants	
Low copay for brand prescription	0.01^***^ [0.00, 0.05]
Monthly premium	0.02^***^ [0.00, 0.11]
No hospital deductible	0.04^***^ [0.01, 0.18]
Access to physicians out-of-network	0.34 [0.07,1.55]

Note. Adjusted associations between health literacy and Medicare Advantage plan choice were estimated using multinomial logistic regression. Plan C is the comparison choice. ADL = activities of daily living; CI = confidence interval; FFS = fee for service; IADL = instrumental activities of daily living.

* *p* < 0.1.

** *p* < .05.

*** *p* < .01.

**Table A:** Ranking Importance of Elements of Health Plan (Exercise 1)

The plan lets me see any doctor I choose The plan has low fees for office visits with a doctor The plan pays all of the cost if I have to stay in the hospital The monthly cost of the plan is very low The plan has low fees for brand name drugs

**Table B:** Frequency of Missingness (*N* = 311)

**Variable**	**Percentage**

Plan choice	14.47
Health literacy	8.04
Chronic conditions	1.93
Insurance preferences	7.07
Age	1.29
Gender	1.29
Race	1.61
Education	1.29
Income	2.89
Insurance	4.5
Health status	6.43
Instrumental activities of daily living	1.93
Activities of daily living	2.25
Annual primary care visits	6.75
Annual specialty care visits	2.89
Number of medications	2.89
Monthly medication costs	0.96
Annual emergency department visits	1.29
Annual admissions to hospital	1.29
Difficulty paying health care costs	2.25

**Table C:** Predicted Probability of Plan Choice by Health Literacy

**Health Literacy Score**	**Intermediate Option Plan A**	**Lower-Premium, Less Coverage Plan B**	**Higher-Premium, More Coverage Plan C**
	**95% CI**		
Adequate health literacy	0.29^*^ [0.21, 0.36]	0.08^*^ [0.04, 0.11]	0.64^*^ [0.56, 0.71]
Marginal health literacy	0.28^*^ [0.09, 0.47]	0.05 [0.5, 0.14]	0.67^*^ [0.47, 0.87]
Inadequate health literacy	0.24^*^ [0.07, 0.41]	0.24^*^ [0.07, 0.40]	0.52^*^ [0.32, 0.72]

Note. CI = confidence interval.

* *p* < .01.

**Table D:** Sensitivity Analysis of Regressions to Combining Marginal and Inadequate Health Literacy Categories (*N* = 311)

	**Plan Preferences**	**Plan A (Medium Cost, Medium Coverage)**	**Plan B (Low Cost, Low Coverage)**
**Characteristic**	**Odds Ratio [95% CI]**	**Relative Risk [95% CI]**	
Health literacy			
Adequate	—	—	—
Marginal/inadequate	0.45^*^ [0.23, 0.90]	0.99 [0.37, 2.66]	2.35 [0.67, 8.22]

Age			
60–69 years	1.38 [0.72, 2.65]	1.54 [0.69, 3.47]	0.61 [0.17, 2.22]
70–79-years	0.85 [0.42, 1.70]	1.94 [0.81, 4.69]	1.16 [0.28, 4.79]
>80 years	—	—	—

Sex			
Male	0.48^*^ [0.26, 0.86]	1.07 [0.53, 2.16]	1.19 [0.37, 3.84]

Race			
White	—	—	—
Black	0.57 [0.29, 1.12]	1.69 [0.70, 4.08]	2.31 [0.55, 9.75]
Other	0.64 [0.33,1.24]	1.82 [0.73, 4.55]	2.88 [0.75, 11.10]

Education			
Less than high school	0.73 [0.35,1.51]	1.46 [0.66, 3.25]	1.35 [0.37, 4.96]
High school	1.42 [0.56, 3.58]	1.62 [0.51, 5.14]	0.27 [0.05, 1.57]
>High school	—	—	

Income			
Low income (≤$2,000)	0.47^*^ [0.25, 0.89]	1.05 [0.49, 2.28]	1.18 [0.35, 3.96]
High income (>$2,000)	—	—	—

Insurance			
Medicare FFS	3.85^**^ [1.64, 9.03]	0.81 [0.30, 2.15]	0.33 [0.06, 1.70]
Medicare Advantage	—	—	—
Supplemental prescription	3.48^**^ [1.78, 6.79]	0.77 [0.36, 1.67]	0.16^*^ [0.03, 0.79]
Supplemental (MediGap)	3.04^**^ [1.22, 7.57]	0.52 [0.15, 1.80]	0.5 [0.08, 2.92]

Health status			
Excellent/very good	—	—	—
Good	0.69 [0.36, 1.33]	1.12 [0.48, 2.62]	0.36 [0.10, 1.31]
Fair/poor	0.62 [0.29, 1.29]	1.21 [0.43, 3.39]	1.14 [0.26, 5.05]

Chronic conditions			
0–1	—	—	—
2–3	0.62 [0.32, 1.21]	0.84 [0.36, 1.94]	1.3 [0.40, 4.19]
4+	0.93 [0.40, 2.15]	0.96 [0.33, 2.79]	0.76 [0.14, 4.10]

Functional health status			
Any ADL impairment	0.94 [0.49, 1.78]	1.4 [0.63, 3.11]	2.44 [0.70, 8.58]
Any IADL impairment	0.53^***^ [0.27, 1.05]	1.94 [0.84, 4.52]	1.77 [0.49, 6.40]

Annual primary care visits			
0–1	—	—	—
2–3	1.21 [0.50, 2.96]	2.19 [0.76, 6.37]	0.76 [0.15, 3.80]
4–5	1.46 [0.57, 3.76]	1.05 [0.34, 3.28]	0.88 [0.17, 4.51]
6+	1.66 [0.59, 4.63]	1.18 [0.33, 4.29]	0.59 [0.08, 4.51]

Annual specialty care visits			
0–3	0.9 [0.45, 1.80]	1.58 [0.63, 3.94]	1.99 [0.49, 8.12]
4–8	—	—	—
9+	1.15 [0.61, 2.15]	1.17 [0.57, 2.41]	1.88 [0.48, 7.28]

Number of medications taken daily			
0–2	—	—	—
3–6	0.89 [0.46, 1.75]	1.09 [0.49, 2.41]	1.47 [0.37, 5.87]
7+	1.26 [0.51, 3.15]	0.88 [0.31, 2.48]	1.21 [0.18, 7.97]

Monthly medication costs			
$0–$9	—	—	—
$10–$49	1.26 [0.64, 2.45]	0.83 [0.34, 2.03]	0.45 [0.11, 1.82]
$50+	0.96 (0.43, 2.13]	0.72 [0.26, 1.99]	0.93 [0.2, 4.07]

Annual emergency department visits			
0–1	—	—	—
2–5	0.87 [0.48, 1.58]	0.61 [0.31, 1.22]	0.82 [0.24, 2.86]
+6	0.52 [0.12, 2.15]	0.59 [0.09, 4.05]	7.88^*^ [0.90, 69.27]

Annual admissions to hospital			
0–1	—	—	—
2–5	0.91 [0.48, 1.72]	1.08 [0.46, 2.52]	2.66 [0.81, 8.70]
6+	0.12^***^ [0.01, 1.14]	5.05 [0.43, 59.15]	0.00^**^ [0.00, 0.01]

Difficulty paying health care costs			
Not at all	—	—	—
Somewhat	0.63 [0.35, 1.16]	0.72 [0.31, 1.68]	1.3 [0.37, 4.53]
Difficult	0.81 [0.22, 2.90]	2.52 [0.79, 8.03]	2.28 [0.22, 23.31]
Very difficult	0.47^***^ [0.20, 1.12]	1.45 [0.43, 4.89]	3.12 [0.69, 14.10]

Constant	—	0.08^*^ [0.01, 0.60]	0.04^*^ [0.00, 0.82]

Constants			
Low copay for brand prescription	0.01^*^ [0.00, 0.04]	—	—
Monthly premium	0.02^*^ [0.00, 0.10]	—	—
No hospital deductible	0.04^*^ [0.01, 0.17]	—	—
Access to physicians out-of-network	0.31 [0.07, 1.39]	—	—

Note. Adjusted associations between health literacy and Medicare Advantage plan choice were estimated using multinomial logistic regression. Plan C is the comparison choice. ADL= activities of daily living; CI = confidence interval; FFS = fee for service; IADL = instrumental activities of daily living.

* *p* < 0.1.

** *p* < .05.

*** *p* < .01.
